# Metallic bands in chevron-type polyacenes†

**DOI:** 10.1039/d0ra06007k

**Published:** 2020-09-14

**Authors:** Mohammed A. Kher-Elden, Ignacio Piquero-Zulaica, Kamel M. Abd El-Aziz, J. Enrique Ortega, Zakaria M. Abd El-Fattah

**Affiliations:** a Physics Department, Faculty of Science, Al-Azhar University Nasr City 11884 Cairo Egypt z.m.abdelfattah@azhar.edu.eg zakaria.eldegwy@gmail.com; b Physik Department E20, Technische Universität München 85748 Garching Germany; c Centro de Física de Materiales CFM/MPC (CSIC-UPV/EHU) 20018 Donostia-San Sebastián Spain; d Donostia International Physics Center 20018 Donostia-San Sebastian Spain; e Departamento de Física Aplicada I, Universidad del País Vasco San Sebastián Spain

## Abstract

We present electronic structure calculations based on a single-parameter plane wave expansion method for basic graphene building blocks, namely *n*-oligophenylenes and *n*-oligoacenes, revealing excellent agreement with density-functional theory. When oligophenylene molecules are joined through *meta* (zigzag) or *ortho* (chevron) junctions, the resulting molecular dimers and polymers exhibit a semiconducting character. While zigzag dimers of oligoacenes also exhibit gapped electronic structures, their chevron-phase features a sharp metallic band at the Fermi energy. This zero-point-energy state, which transforms into Dirac-like band in chevron polymers, survives at the outer elbows of the dimer irrespective of the molecular length, and has the same origin as reported for the polyacetylene and topologically induced edge states at edge-decorated graphene nanoribbons. These findings assist the engineering of topological electronic states at the molecular level and complement the toolbox of quantum phases in carbon-based nanostructures.

## Abstract 2

We present electronic structure calculations based on a single-parameter plane wave expansion method for molecular nanostructures revealing excellent agreement with density functional theory and predicting metallic bands for chevron molecular dimers.

## Graphical abstract

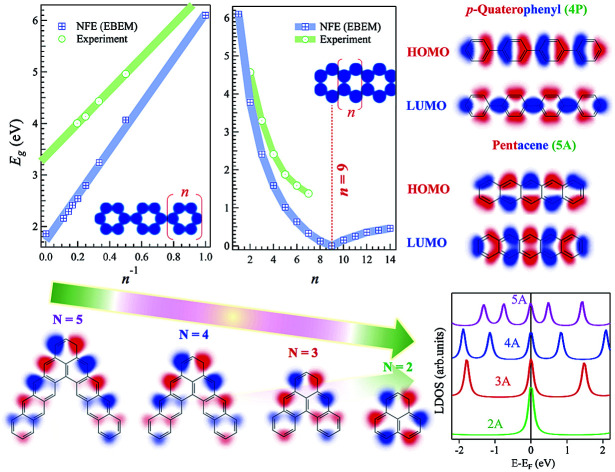

## 1 Introduction

The crossover from three-dimensional (3D) graphite to lower dimensions has enabled the exploration of exotic phases of matter hosted by these carbon nanostructures. Starting from the pioneering exfoliation of graphene,^1,2^ an atomic 2D honeycomb carbon lattice, and by virtue of its unique conical dispersion,^3,4^ a wide range of quantum phenomena^5,6^ and potential devices^7,8^ based on relativistic Dirac-fermions have emerged. Likewise, the bottom-up on-surface chemistry route facilitates the production of 1D carbon nanostructures, namely graphene nanoribbons (GNRs), with tailored properties that are tunable through width, shape, and edge terminations.^9–11^ For example, zigzag GNRs (ZGNRs) featured a playground to explore edge physics and sp-type magnetism, armchair GNRs (AGNRs) enabled the formation of atomically tunable band gaps required for semiconducting devices, while edge-corrugated GNRs offered a customizable platform towards the exploration of topological states in carbon structures.^12,13^ Furthermore, the GNRs width has been pushed down to the extreme limit, where the narrowest semiconducting poly(*para*-phenylene) polymer (3-AGNRs)^14^ and the single carbon atom wide metallic polyacetylene^15^ have been successfully produced. The degree of electron delocalization within these ultimately narrow structures could be tuned by controlling the edges and/or linkers topology^16,17^ leading to distinguished electronic properties such as the formation of unconventional indirect band gaps within these polymers.^18^

Nowadays, the down scaling of these 1D structures into graphene nano-dots and aromatic graphene-based large molecules, as 0D carbon structures, is gaining further interest aiming to transfer the inherited graphene and GNRs physics to the smallest scale possible. With the current advance in chemical methods and by selecting the right molecular precursor, a number of such 0D graphene structures are made possible. For instance, large polyacene and zigzag-edged triangular graphene molecules which are predicted to host edge-ferromagnetism suitable for next-generation molecular spintronics, are successfully produced following the on-surface chemistry approach.^19–26^

This revolutionary advance of the bottom-up on-surface chemistry calls *a priori* for theoretical tools for the proper selection and prediction of carbon structures with on-demand tailored properties. Density-functional theory (DFT) and nearest-neighbor (NN) tight binding (TB) model^27^ are the essential tools. However, with the reduced dimensionality from 2D graphene into 1D GNRs, NN-TB fails to capture relevant electronic structure details. For example, while a second NN hopping parameter is required to account for electron–hole asymmetry in extended graphene,^4,28^ the braiding of valence and conduction bands in narrowest ZGNRs calls for a third NN hopping parameter.^29^ These limitations are likely to become more pronounced for 0D systems given that the number of NN hopping parameters becomes of the same order of magnitude as the molecular length scale. Recently, a single-parameter electron plane wave expansion (EPWE) model was proposed to describe, within DFT level of accuracy, the electronic structure of both 2D graphene and 1D GNRs alike.^30^ The model captures all possible cross-talks between neighbor carbon-atoms thereby offering a uniquely simple approach suitable for the prediction of the electronic structure of small carbon-based nanostructures.

Here, we first demonstrate that the EPWE picture applied to graphene and GNRs, can be extended to describe the π-electronic characteristics of 0D carbon structures, such as oligophenylene and oligoacenes. The valence and conduction bands braiding for the narrowest ZGNRs^29^ and the gap size oscillation reported for *n*-oligoacenes^31,32^ are well reproduced from our single parameter (*i.e.*, a scattering potential) calculations. Second, we show the applicability of this model to describe molecular junctions forming zigzag-like or chevron-like dimers and polymers. We identify sharp metallic bands at the Fermi energy, exclusively for chevron-phase oligoacenes, which are present irrespective of the molecular length. This band bears the same origin as those reported for the toy-model polyacetylene and for the emerging topological edge states in decorated GNRs. These findings demonstrate that our simple EPWE framework is well suited for 0D carbon-nanostructures and has the potential to predict peculiar properties associated with graphene-based molecular structures of complex architectures.

## 2 Methods

**Fig. 1:**
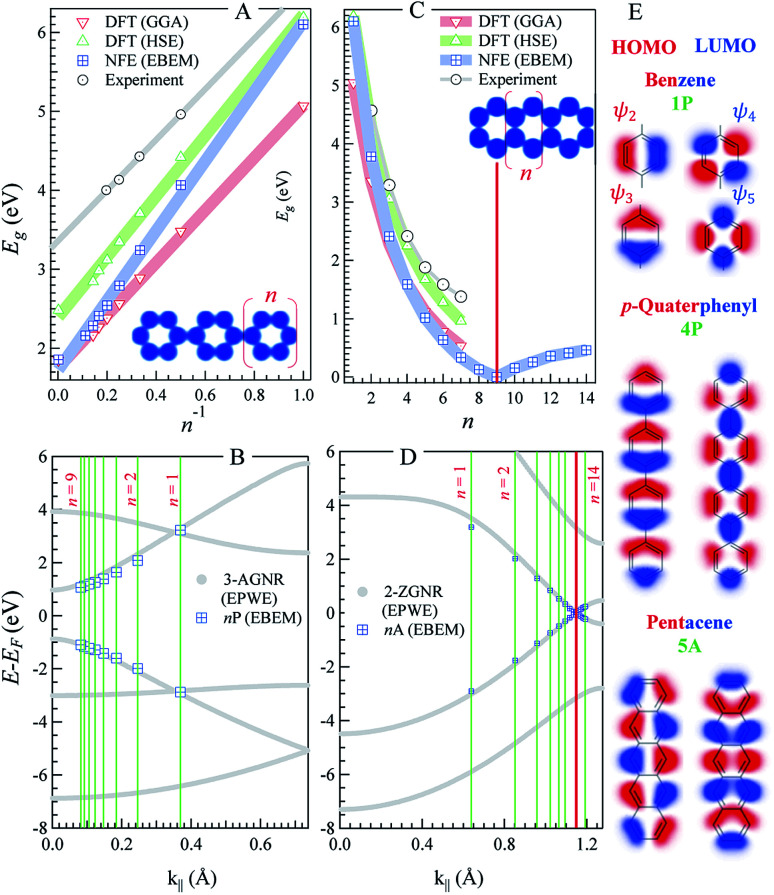
*n*-oligophenylenes *vs. n*-oligoacenes. (A and C) The HOMO–LUMO separation (*E*_g_) calculated within our EBEM (blue) approach for *n*-oligo-phenylenes (A) and *n*-oligoacenes (C) molecules as a function of the molecular length (*n* being the number of fused phenyl rings). DFT calculations using GGA (red) and HSE (green) functionals as well as experimental data (grey) are extracted from ref. 35 and 36, respectively, and presented for comparison. (B and D) EPWE calculated band structures for the corresponding infinite polymers, *i.e.*, *n*-poly(*para*-phenylene) or 3-AGNR (B) and *n*-polyacenes or 2-ZGNR (D).The red lines in (C) and (D) mark the gapless molecular length and the 2-ZGNR Dirac-point degeneracy, respectively. (E) The HOMO and LUMO positions obtained from EBEM (blue) are appended in (B) and (D) at the momenta marked by green lines [π/(*n* + 1)*d*]. (E) Molecular orbitals taken at the HOMO and LUMO position for benzene (top), *p*-quaterphenyl (middle), and pentacene (bottom).

We simulate the electronic structure of these molecular systems by defining 2D potential landscapes, where each carbon atom is represented by a circle filled with uniform zero-potential embedded in a flat interstitial region of potential *V* ≈ 23 eV [blue and white regions in the insets of Fig. 1(A and C)]. We write the Schrödinger equation as1
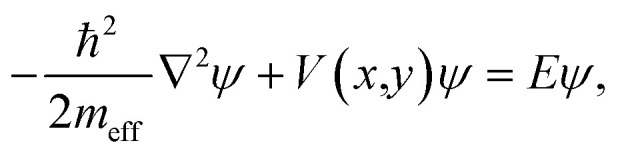
where the energy *E* is expressed relative to a reference level (*e.g.*, the Dirac point of 2D graphene), *m*_eff_ is the effective mass (here, *m*_eff_ = *m*_e_), *V*(**R**) is the 2D potential as a function of spatial coordinates **R** = (*x*,*y*), and *ψ*(**R**) is the electron wave function. We then solve eqn (1) using either an electron-plane-wave expansion (EPWE) or boundary element method (EBEM) implementations, respectively, for extended and finite systems, as detailed in ref. 30, 33 and 34. For molecules and polymers calculations using EPWE we employ the super cell approach separating both molecules (along *x*- and *y*-directions) and polymers (along *y*-direction) by distances ≥ 10 Å, and terminating the potential expansion at *g*_max_ ≥ 20, where *g*_max_ refers to the maximum number of reciprocal lattice vectors relative to the origin of the reciprocal space.^30^

## 3 Results and discussion

Fig. 1 depicts the electronic characteristics of *n*-phenylenes (*n*P) and *n*-acenes (*n*A) molecules as determined from the EBEM (blue) approach. Their potential landscapes and the building units are shown in the inset of (A and C). In (A) the energy gap (*E*_g_) defining the separation between highest occupied and lowest unoccupied molecular orbitals (HOMO and LUMO) is plotted for *n*P molecules as a function of the number of phenyl rings (*n*). We note that *E*_g_ follows a linear relation with (1/*n*) in agreement with DFT calculations obtained with (red) generalized gradient approximation (GGA) and (green) the range-separated hybrid functional (HSE).^35^ We note that the experimentally measured band gaps (grey)^36^ are wider than both EBEM and DFT calculations, being closer to the HSE results, suggesting a significant electron–electron correlation effects which are not properly considered in GGA and, obviously, in our single-electron EBEM calculations. In this context, *GW* approximation has shown to provide more accurate quasiparticle band gap for graphene nanostructures, which can be larger than ∼3 eV compared to conventional DFT calculations.^37^ We obtain exactly the same EBEM results by employing EPWE approach for molecules separated by 10 Å along *x*- and *y*-directions. In what follows, we use EBEM for finite molecules, while restricting EPWE calculations for their corresponding extended polymer structures, unless otherwise specified. Fig. 1(B) presents the band structure (grey) for the infinite *n*P polymer, namely poly(*para*-phenylene) or equivalently 3-AGNR. The energetic positions of the first HOMOs and LUMOs of *n*P molecules obtained from EBEM (blue) are inserted at momenta values of π/(*n* + 1)*d* as indicated by the green vertical lines (
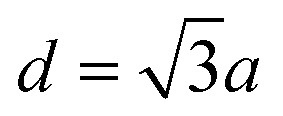
, *a* = 1.42 Å is the carbon–carbon distance). It is clear that the HOMOs and LUMOs follow precisely from the frontier valence and conduction bands of 3-AGNR, respectively. Indeed, the energetic positions of all *n*P molecular orbitals can be obtained following this slicing scheme of the Brillouin zone (BZ), similar to the extraction of AGNRs electronic structure from the 2D graphene bands [see ESI, Fig. S1†].

In Fig. 1(C) the *E*_g_*vs. n* plot for *n*A molecules obtained from EBEM, GGA-DFT, and HSE-DFT calculations is presented. The gap size reduces faster than the case of *n*P molecules until it eventually closes for *n* = 9 molecules. The metallicity of this 9A molecule can be assured from the presence of a sharp peak in the corresponding LDOS at the Fermi energy [see ESI, Fig. S2†]. For *n* ≥ 10, small gaps open up, in agreement with the gap size oscillation reported from recent DFT calculations.^38,39^ We note that the presence of structural instabilities or electron–electron interactions have the effect of lifting up this degeneracy. Gap closing and reopening are understood from a closer inspection into the band structure of the extended (*n* = ∞) polyacene (2-ZGNR) presented in (D). This narrowest ZGNR exhibits an edge state with lifted degeneracy (∼0.86 eV gap at BZ boundary) except at a single noncommensurate momentum at the vertical red line (*k*_∥_ ∼ 1.15 Å^−1^), where the Dirac-like degeneracy point is kept. It is the presence of this degeneracy and the braiding of the valence and conduction bands that are responsible for the closing and reopening of the energy gaps, respectively [see ESI, Fig. S3†]. The length of the molecule at which the gap closes is extremely sensitive to this momentum position, where the gap closes for *n* = 10 molecule when the degeneracy is found at *k*_∥_ ∼ 1.16 Å^−1^.^38^ It is worth noting that such edge state braiding effects do not show up in standard NN-TB models, and are resulting from the inclusion of the third hopping parameter, reassuring that our single-parameter nearly free electron approach intrinsically captures higher order hopping(s), leveling to *ab initio* DFT results.^38,39^ Finally, we present, in Fig. 1(E), the spatial distribution of the wave functions (*i.e.*, the 2D orbitals shape) taken at the first HOMO (red) and LUMO (blue) energies, for three selective molecules; benzene, *p*-quaterphenyl (4P), and pentacene (5A). For instance, the HOMO of 5A molecule exhibits five lobes and a nodal plane, along the edges and molecular axis, respectively, while the LUMO contains two end lobes enclosing five axial lobes, in good agreement with the molecular orbitals obtained from DFT calculations.^40^ Notice the presence of spectral intensity at the bonds position and the non-spherical deformation of the orbitals at carbon atoms, both demand higher order overlap integrals in TB calculations which are often neglected.

From the above discussion we demonstrated the capability of our simple nearly-free-electron model to well describe the electronic characteristics of oligophenylenes and oligoacenes molecules as simple graphene and GNRs building units. Also the fine electronic details of the thinnest ZGNRs, with characteristic valence and conduction bands braiding and noncommensurate Dirac-point, are well reproduced.

**Fig. 2:**
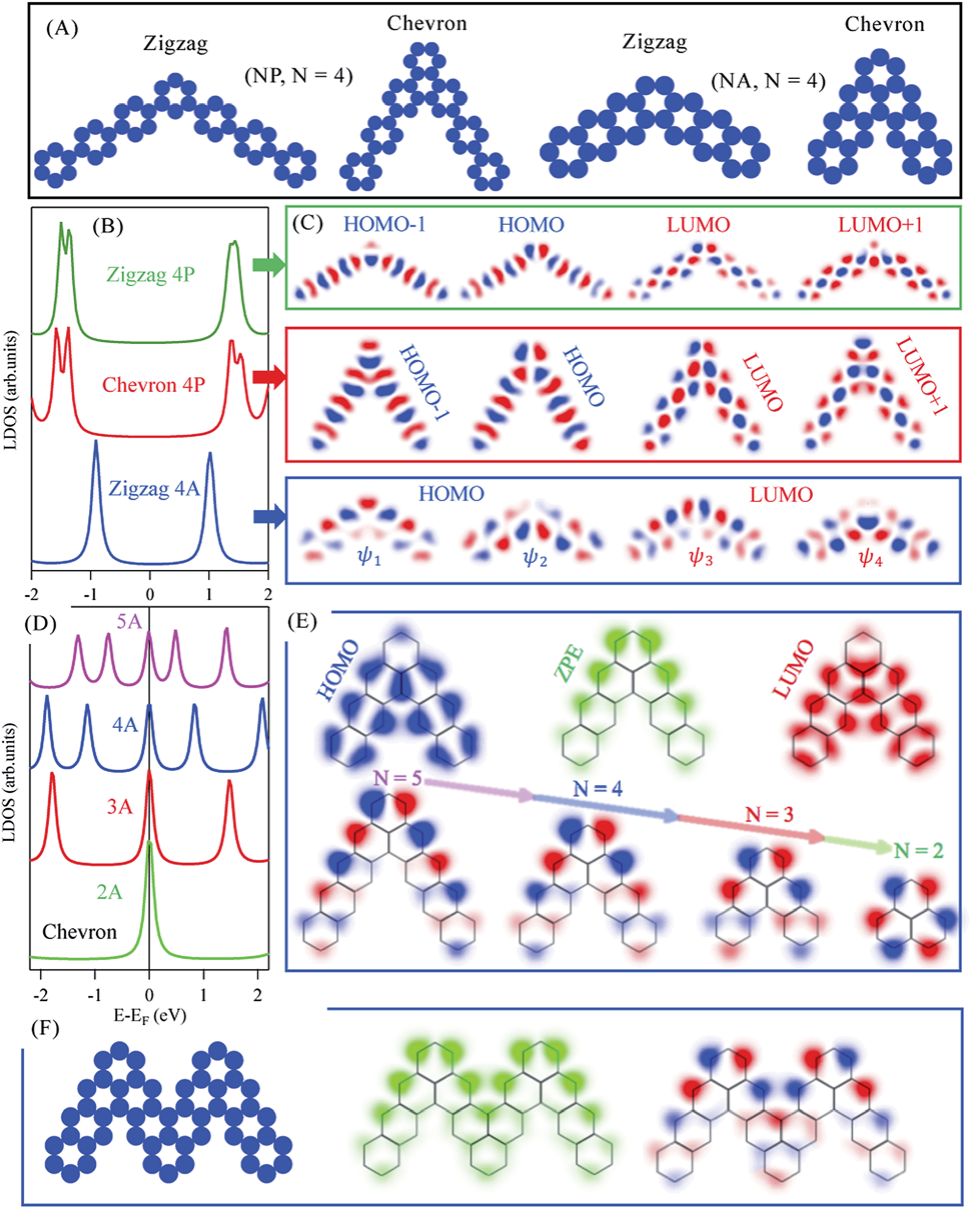
*N*P and *N*A molecular dimers. (A) The potential landscapes used as EBEM inputs for the zigzag and chevron phases of *N*P and *N*A (*N* = 4) kinked isomers. (B) The LDOS for the zigzag (green) and chevron (red) phase of 4P and for the zigzag 4A phase (blue). (C) The spatial distribution of wave functions for the first and second HOMOs and LUMOs of the three cases in (B). (D) The LDOS for the chevron phase of *N*A molecules for different *N* values. (E) Top: The spatial distribution of DOS taken at the HOMO, LUMO, and at the zero-point-energy (ZPE, Fermi level) for the case of 4A chevron phase, and Bottom: the wave functions at ZPE for different *N* values. (F) Left: The potential landscape for two joined kinked 4A isomers forming chevron phase and the corresponding LDOS (middle) and wave function (right) taken at ZPE.

Molecular junctions and topologically corrugated polymers based on such graphene building blocks are gaining growing interest both from experimental and theoretical view points, as they offer additional means of precisely engineering structures with on-demand emerging electronic properties.^17,41–43^ In the following we study the electronic structure evolution for *N*P and *N*A molecular dimers connected through *meta* and *ortho* junctions. We refer to these junctions as zigzag and chevron phases with 120° and 60° between arms, respectively, and define *N* as the number of benzene rings per molecular arm, as sketched in Fig. 2(A). The fabrication of such molecular dimers was recently achieved following on-surface chemistry.^16,20,44^Fig. 2(B) presents the LDOS, integrated over all carbon atoms within each molecular dimer, for the zigzag (green) and chevron (red) phases of 4P molecular dimers. Both structures exhibit semiconductor character with slightly larger (Δ*E*_g_ ∼ 0.35 eV) HOMO–LUMO gaps than the corresponding straight (*para*) 7P molecule, indicating partial electrons localization at the junctions (elbows) position. The first and second HOMOs and LUMOs of these structures are slightly energy-split and are composed of the 3P molecule and elbows induced orbitals at junctions position, Fig. 2(C). The overall wave functions and their fine spatial variations at elbows here reported are in perfect agreement with recent experimental and DFT calculations for the *N*P zigzag structures.^16^ Likewise, the zigzag phase of 4A molecular dimer is a semiconductor with significantly larger (Δ*E*_g_ ∼ 1.75 eV) gap compared to the corresponding heptacene (7A) molecule. The first and second HOMOs and LUMOs in this case are nearly degenerate, Fig. 2(B), with wave functions localization at either sides of the dimers including the elbows, Fig. 2(C). The overall electronic structure of these three families follow from their corresponding extended polymers, shown in ESI, Fig. S3,† which exhibit clear semiconductor band structures with similar dispersions for zigzag and chevron *N*P dimers. We note that the incorporation of these junctions, intuitively, increases the semiconducting character compared to the corresponding straight molecules/polymers, irrespective of the molecular family (*n*-oligophenylene or *n*-oligoacenes) or the junction type (zigzag or chevron), since electrons cross-talk is broken at junction positions.

Of special interest is the chevron-phase of *N*-oligoacenes dimers whose LDOS are presented in Fig. 2(D). The LDOS features a zero-point-energy (ZPE) state pinned at the Fermi energy irrespective of the length of these dimers, in addition to the common HOMOs and LUMOs levels with reduced separation for longer dimers. Notice that the first HOMO and LUMO for this chevron-type 4A dimer, for example, are practically coincident with the corresponding zigzag phase presented in Fig. 2(B, blue), except for the ZPE state in the former. The 2D-LDOS at the first HOMO (blue) and LUMO (red) as well as at the ZPE (green) for the chevron-type 4A molecular dimer are given in Fig. 2(E, top). While the LDOS of the HOMO and LUMO greatly resemble those obtained for individual 3A molecules, with vanishing contribution at junctions, the ZPE state exhibits strong localization at the elbow and the outer edges of the dimer (green). In Fig. 2(E, bottom) we present the spatial distribution of the wave functions at this ZPE for chevron dimers of different length. The wave function is clearly localized at the outer edge for all molecules, and eventually becomes symmetrically distributed at the outer carbon-sublattice for the smallest 2A molecule, namely phenalenyl. Notice that the ZPE here reported for phenalenyl radical is the same singly occupied molecular orbital (SOMO) obtained from early Hückel calculations with its characteristic hexagonal arrangement at one-lattice type, being one of the classical building units towards the exploration of stacked metallic carbon structures and molecular spintronics.^45–47^ In fact all longer chevron *N*A dimers are composed of such phenalenyl radical at their elbows joined through *ortho* junction with *N*-2 benzene ring per molecular arm, similar to recently proposed open-shell graphene fragments, namely benzo[*cd*]triangulenes.^48^ For such longer dimers, the same SOMO state is mainly localized at one outer sublattice with minor contribution at the same inner carbon-sublattice. This clear distinction between the metallic chevron and semiconducting zigzag dimers can be inferred from the benzenoid graph theory, which predicts the number of ZPE states associated with benzenoid structures following a simple counting rule.^49,50^ The number of such ZPE states is given as 2*α* − *ν*, where *α* is the maximum possible number of non-adjacent sites and *ν* the total number of sublattice sites (*ν*_A_ + *ν*_B_). For all *N*A molecules *ν*_A_ − *ν*_B_ = 0 or 1 for zigzag or chevron dimers, respectively, while *α* = *ν*_A_ = *ν*_B_ (zigzag) or *α* = *ν*_A_ = (*ν*_B_ + 1) (chevron). Therefore, irrespective of the arms length, all molecular dimers support a single ZEP state according to this counting rule. The presence of this ZPE state and its edge localization suggest possible formation of coupled and propagating elbow states for fused dimers, analogous to the experimentally released metallicity in GNRs through such zero modes.^51^ The potential landscape of two fused chevron 4A molecular dimers is depicted in Fig. 2(F, left). This large molecule also exhibits a ZPE state, the 2D-LDOS (middle) and wave function (right) of which are given in (F). The coupling between the elbow states is clear and suggests a distinct metallic character of their extended chevron-type polymers.

**Fig. 3:**
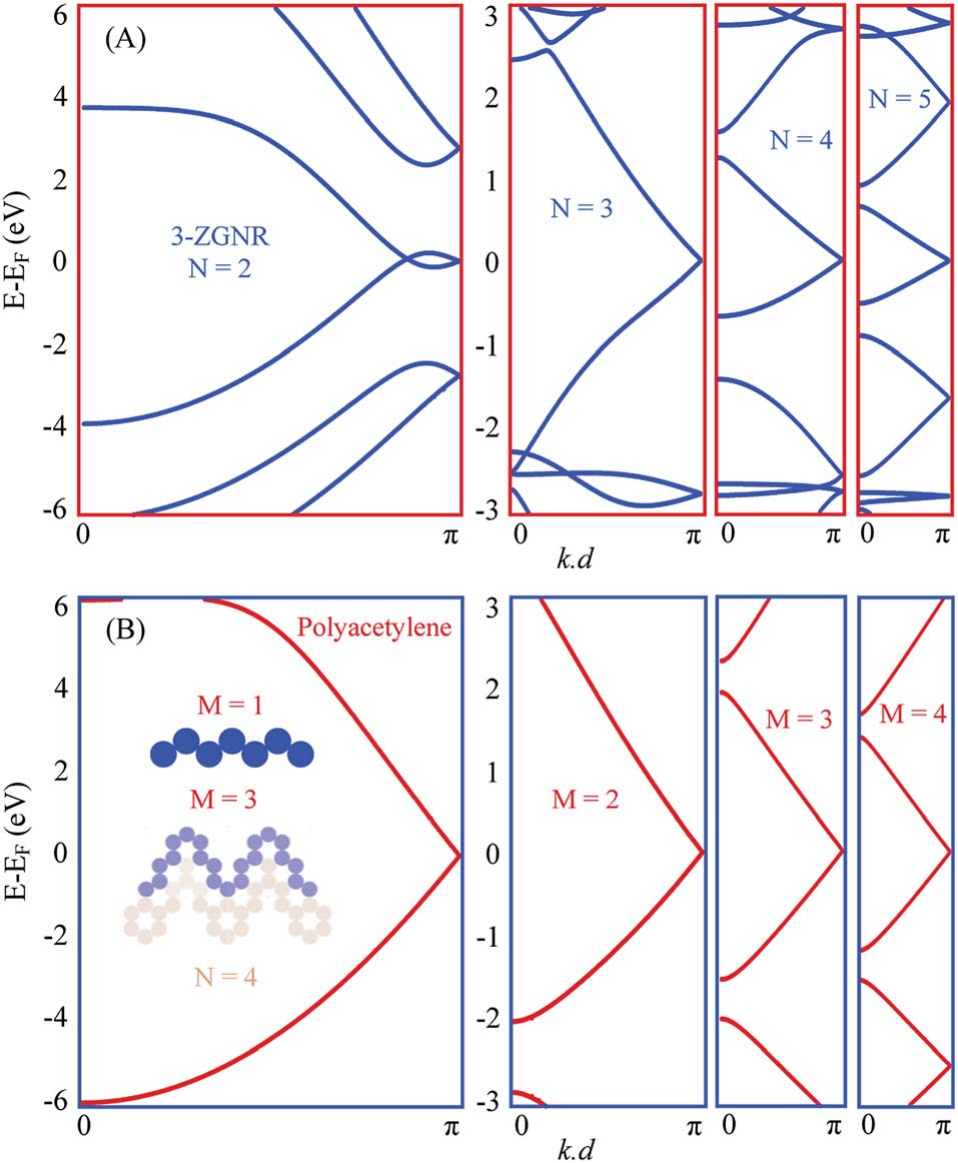
Chevron oligoacene polymer *vs.* polyacetylene. (A) Band structure of *N*A chevron-type polymers for *N* = 2–5. The *N* = 2 polymer is equivalent to 3ZGNR. (B) Band structure of polyacetylene (left) and its chevron-type (right) for *M* = 2–4, where *M* defines the number of carbon dimers per arm. The inset of (B) presents the potential landscape for the straight polyacetylene (*M* = 1) polymer and the corresponding *M* = 3 chevron-type (blue), which defines the edge of 4A chevron-type oligoacene shown in light brown.

In Fig. 3(A) we present the EPWE calculated band structures for such *n*-oligoacene-based chevron polymers with *N* = 2–5. The *N* = 2 polymer, with phenalenyl radical as the building unit, is structurally coincident with the 3-ZGNR. Similar to the metallic band structure of 2-ZGNR depicted in Fig. 1(D), the 3-ZGNR exhibits valence and conduction band braiding with two degenerate Dirac-points; a commensurate one at the BZ boundary and other incommensurate degeneracy at *k*_∥_ ∼ 1.1 Å^−1^, in agreement with DFT and third NN-TB calculations.^29^ This metallic band structure is preserved for chevron polymer of higher *N*, but with a single commensurate degeneracy at BZ boundary. The effect of different *N* values only shows up as relatively flatter dispersion (*i.e.*, smaller Fermi velocity *v*_F_) for polymers with longer arms, yet the degeneracy point is unaltered. Notice that these chevron polymers can be considered as model systems for the Su–Schrieffer–Heeger (SSH) model,^52^ which here, in the absence of arms asymmetry or different hopping parameter per arm, are fully metallic. A toy-model candidate for SSH formalism is the polyacetylene, the EPWE band structure of which is depicted in Fig. 3(B). Given the iso-separation between carbons, shown in the potential landscape in the inset of (B), and the absence of hopping asymmetry, the band structure of this non-dimerized *trans*-polyacetylene exhibits a degeneracy at the BZ boundary, in agreement with DFT calculations.^53^ Notice the obvious asymmetry between valence and conduction bands, which is absent in NN-TB calculations.^15^ Recently, the Peierls instability responsible for bond alternation (*i.e.*, dimerization) is suppressed for highly doped polyacetylene grown onto Cu(110), where the underlying metallic band structure was experimentally identified.^15^ Our intention to present the polyacetylene case into discussion here is simply because it is structurally identical to the edges of polyacene, and when shaped into chevrons it defines the edges of the corresponding chevron *N*A polymers present in (A). The right panels of Fig. 3(B) display the band structures of such chevron-type polyacetylene made of *M* carbon dimers per arm. The potential landscape for *M* = 3, for example, is shown in blue at the inset of (B). It clearly defines the outer edge of the corresponding *N* = 4 chevron oligoacene polymer shown in light-brown. The band structures are clearly metallic for all these chevron-type polyacetylene (B), and greatly resemble those presented in (A).

Notice that this metallic character of the chevron polymers presented in (A) is of significant importance for the realization of topological states in carbon structures. A simple example is the topological end states reported for the nearly metallic 5-AGNR after breaking the nearest-neighbor (NN) hopping symmetry.^54^ The present system offers two different means of symmetry breaking, namely the NN sublattice symmetry for each arm, in addition to the arms symmetry, which can be broken by compression/stretching of the dimer/polymer using, *e.g.*, substrate incommensuration or kinked surfaces as templates. This metallicity which places the system at the border of topological transition, together with the different symmetry breaking mechanisms and their combinations can, therefore, push the system into the topologically nontrivial electronic regime.^55,56^

Despite the growing interest in the exotic properties offered by the metallic phases here discussed, the instability of these and similar open-shell structures including zigzag graphene nanoribbons, acenes, and triangulene is a major concern. Their high chemical reactivity, arising from unpaired electrons contributing near the Fermi energy, renders their synthesis *via* solution-based chemistry rather challenging. During the last few years the surface-assisted synthesis of several prominent types of zigzag-edged nanographenes and nanoribbons have been achieved, while their solution-based chemistry still lags behind due to their poor chemical stability. Therefore, it has been demonstrated that on-surface chemistry in UHV conditions provides the required environment to stabilize graphene nanostructures which lack stability in solution-based chemistry.^57–59^ Following such a powerful on-surface generation method, and through the rational synthesis of precursor molecules, most of the systems here explored can be generated. For instance, the well-established gold(i)-catalyzed cyclization methodology combined with on-surface synthesis has enabled the dehydrogenation of hydrogen-protected acenes on surfaces and has provided an entry into the whole series of higher acenes up to undecacene.^60^ In addition, dodecacene molecules have also been synthesized on Au(111).^61^ The pentaepoxy derivatives, which are obtained by a four-step iterative sequence of aryne cycloadditions in solution, are first deposited on Au(111) and after a thermal deoxygenation, produce dodecacene. Cycloaddition processes can also be achieved on-surfaces, allowing the combination of acenes into long nanoribbons or 2D covalent organic frameworks. Recently, the incorporation of cyclobutadiene moieties (four member rings) as linkers between the acene segments has been reported. These structures are achieved through the formal [2 + 2] cycloaddition reaction of *ortho*-functionalized tetracene precursor monomers.^62,63^ Following this method, in-line tetracene-based nanoribbons connected by cyclobutadiene moieties through formal [2 + 2] cycloaddition of *ortho*-tetrahalogen-functionalized precursor monomers has also been realized.^64^ Therefore, formation of the metallic chevron oligoacene polymers here reported might also be achieved by a similar cycloaddition process. However, we anticipate that their synthesis demands, instead of 4-membered rings (cyclobutadiene moieties), the formation of the more complex 6-membered rings.^65,66^

## 4 Conclusions

In conclusion, we presented a single-parameter electron plane wave expansion (EPWE) approach capable of describing the electronic structure of *n*-oligophenylenes and *n*-oligoacenes molecules with a DFT level of accuracy. Likewise, the model describes the electronic characteristic for ultimately narrow ZGNR and the single-atom wide polyacetylene. Within the same framework, we identified metallic bands presented, exclusively, at the elbows of finite chevron-type molecular dimers made of *n*-oligoacenes or polyacetylene, which transform into Dirac-like bands for their corresponding extended polymers. These findings place the EPWE approach as an efficient tool suitable for understanding and predicting the emerging physics in carbon-nanostructures, such as topological electronic states at the molecular level.

## Conflicts of interest

There are no conflicts to declare.

## Supplementary Material

RA-010-D0RA06007K-s001

RA-010-D0RA06007K-s002
