# Supplementary material for: Metallic bands in chevron-type polyacenes
Source: RSC Adv. 2020 Sep 14;10(56):33844–50. doi: 10.1039/d0ra06007k (PMC9528856; doi:10.1039/d0ra06007k)
Supplement: RA-010-D0RA06007K-s002 [file RA-010-D0RA06007K-s002.zip › SI/Molecules_SI.pdf]

RSC Advances

Electronic supplementary information for **Metallic Bands in Chevron-Type Polyacenes**

Mohammed A. Kher-Elden,[a] Ignacio Piquero-Zulaica,[b] Kamel M. Abd El-Aziz,[a] J. Enrique Ortega,[c,d,e] and Zakaria M. Abd El-Fattah*[a]

[a] *Physics Department, Faculty of Science, Al-Azhar University, Nasr City, 11884 Cairo, Egypt.*
[b] *Physik Department E20, Technische Universität München, 85748 Garching, Germany.*
[c] *Centro de Física de Materiales CFM/MPC (CSIC-UPV/EHU), 20018 Donostia-San Sebastián, Spain.*
[d] *Donostia International Physics Center, 20018 Donostia-San Sebastian, Spain.*
[e] *Departamento de Física Aplicada I, Universidad del País Vasco, San Sebastián, Spain.*

* *Corresponding author: z.m.abdelfattah@azhar.edu.eg, zakaria.eldegwy@gmail.com*

1 Band Structure of 6P Molecule from EPWE and Quantized 3-AGNR

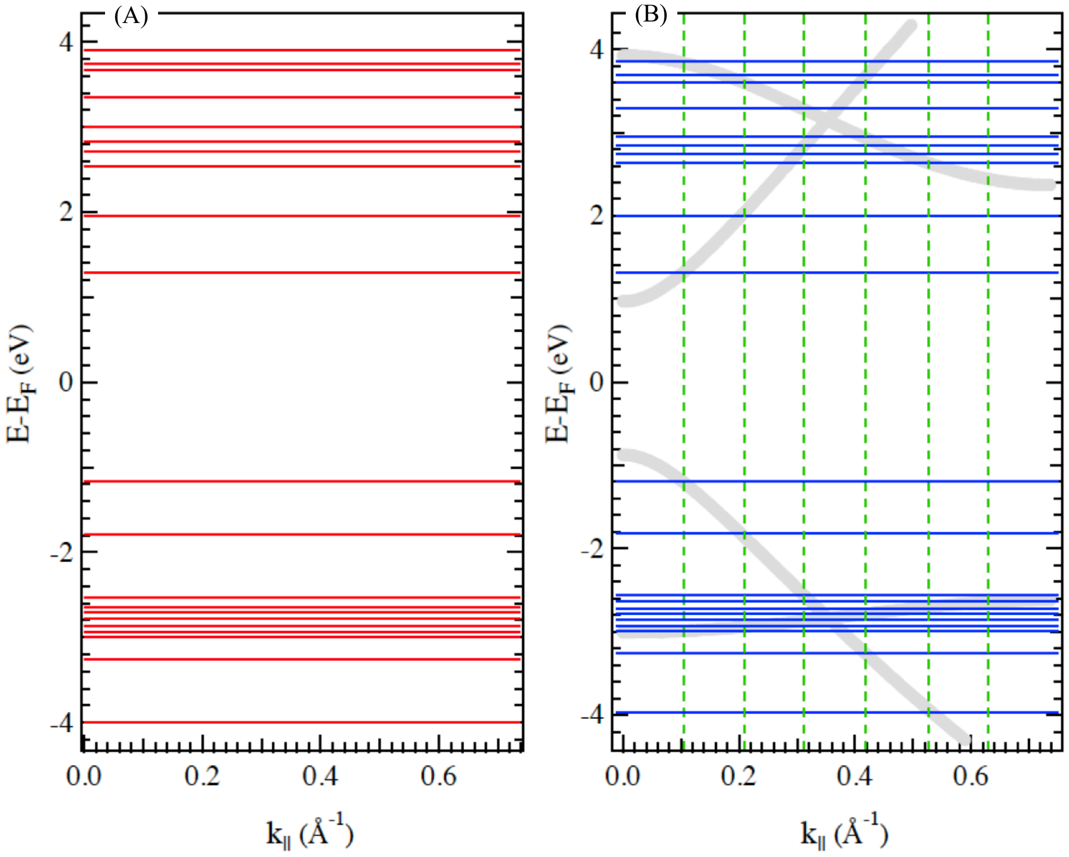

Fig. 1: **p-Sexiphenyl vs. 3-AGNR:** (A) EPWE calculated band structure for 6P molecule, namely p-Sexiphenyl. (B) EPWE calculated band structure for n-poly(para-phenylene) or 3-AGNR (grey). The molecular levels (blue) are obtained by slicing the 3-AGNR band structure at the momenta marked by green dashed lines $[\pi/(n+1)d]$, where $n = 6$ is the number of fused phenyl rings and d is the periodicity along 3-AGNR axis (3×1.42 Å).

2 Density of States for Metallic Oligoacenes

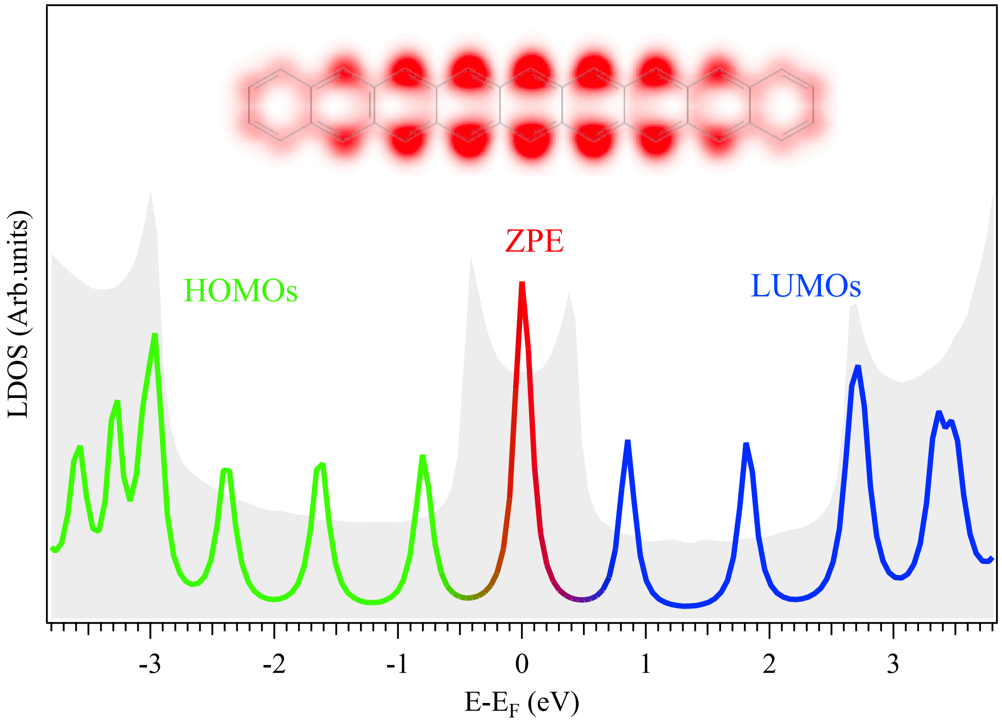

Fig. 2: **Density of States for 9A Molecule and 2-ZGNR:** LDOS for the 9A molecule, i.e., oligoacenes of $n = 9$, showing the typical HOMOs (green) and LUMOs (blue) in addition to the emerging metallic ZPE state (red). The grey area denotes the DOS of the infinite polyacene polymer, i.e., the 2-ZGNR, with the typical U-shape LDOS near the Fermi energy[1]. The inset presents 2D-LDOS map, taken at the ZPE, showing the delocalization of the ZPE state at the outer edges of the 9A molecule with intensity that decays from its maximum at the molecular center towards the ends in agreement with DFT calculations[2].

3 EPWE vs. EBEM Comparison and the Gap Size Oscillation in Oligoacenes

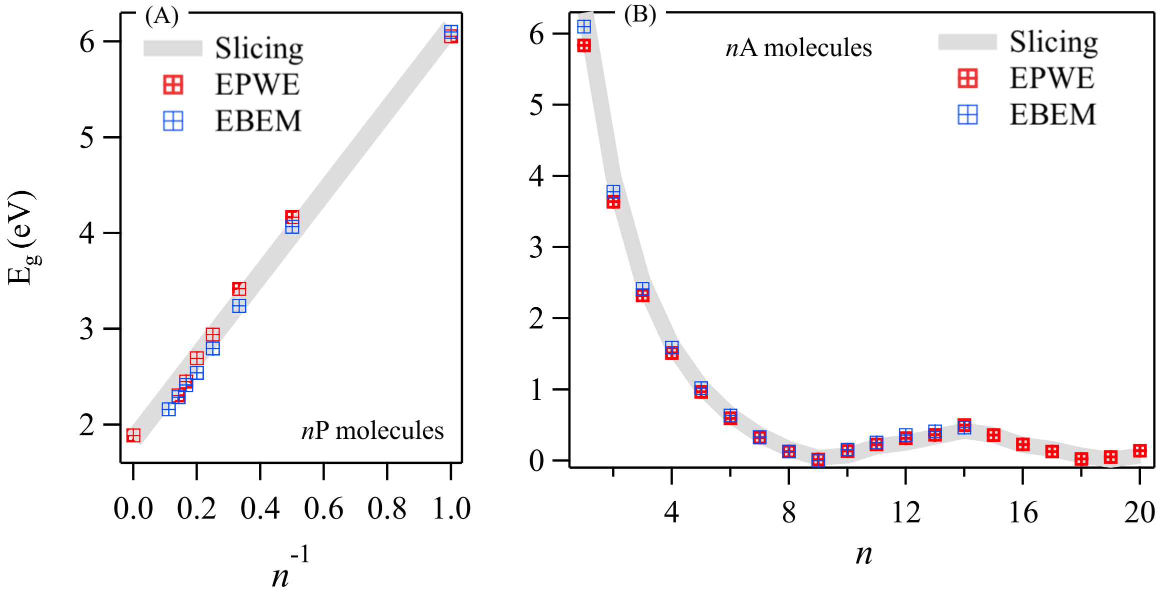

Fig. 3: **EPWE vs. EBEM:** . (A,B) The HOMO-LUMO separation (E_g) calculated within EPWE (red) and EBEM (blue) approaches for n-oligo-phenylenes (nP) and n-oligoacenes (nA) molecules as a function of the molecular length (n being the number of fused phenyl rings). The grey curves in (A) and (B) correspond to E_g obtained by slicing the band structures of 2-ZGNR and 3-AGNR, respectively, at the momenta given by $\pi/(n+1)d$. The overall agreement between the extended EPWE and finite EBEM calculations is remarkable, while the gap size oscillation in (B) agrees with DFT calculations[3,4].

4 Semiconducting Zigzag and Chevron Polymers

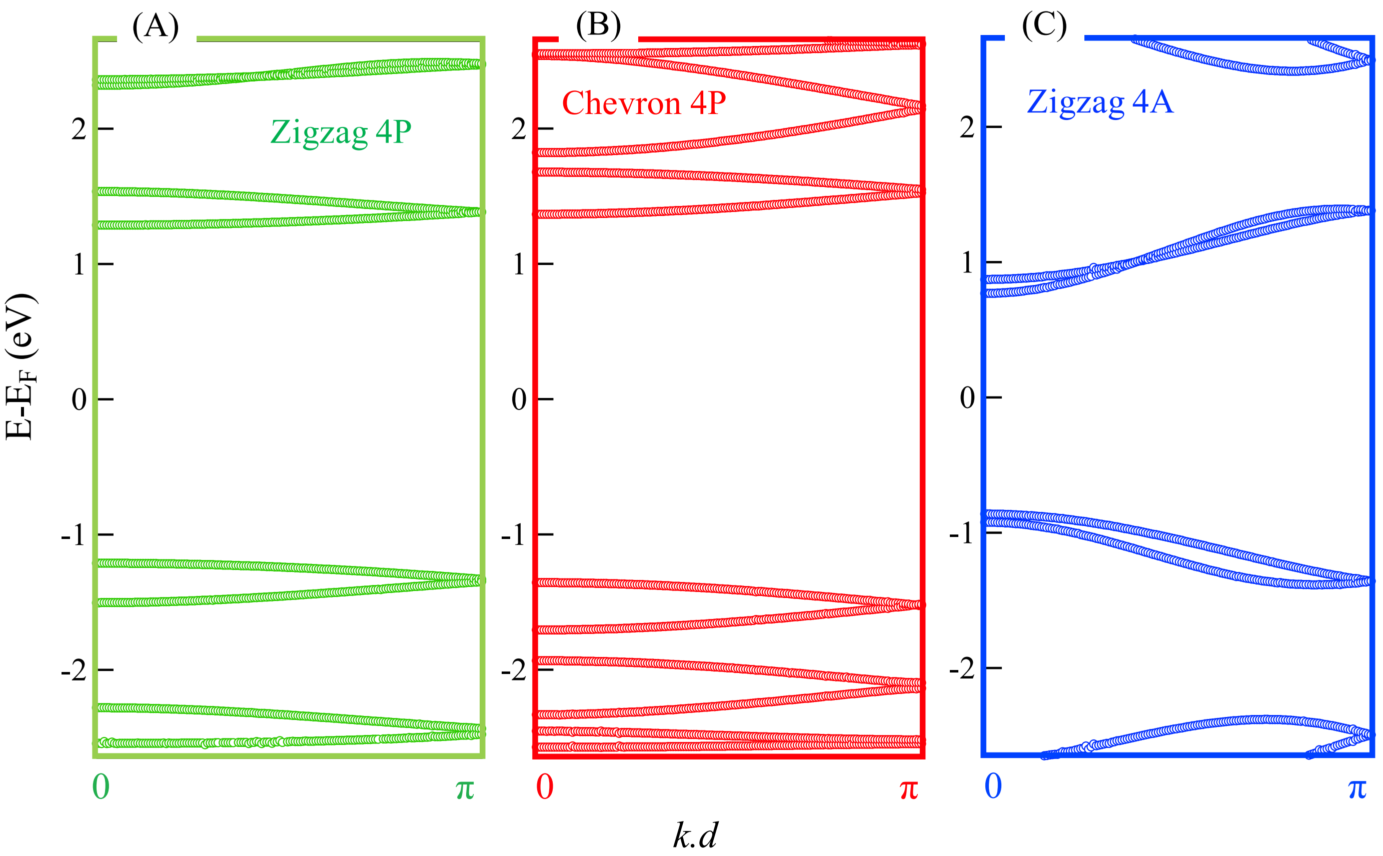

Fig. 4: **Semiconducting Polymers:** EPWE calculated band structures for the zigzag and chevron 4P polymers (A and B) and for the zigzag 4A polymer (C), where all polymers are semiconductors. The band gap for the zigzag 4P polymer (A) is $\sim$ 2.5 eV in agreement with DFT calculations [5].

References

1 Correa, J. H.; Pezo, A.; Figueira, M. S. Braiding of edge states in narrow zigzag graphene nanoribbons: Effects of third-neighbor hopping on transport and magnetic properties. *Phys. Rev. B* **2018**, *98*, 045419.

2 Huang, R.; Phan, H.; Herng, T. S.; Hu, P.; Zeng, W.; Dong, S.-q.; Das, S.; Shen, Y.; Ding, J.; Casanova, D.; Wu, J. Higher Order -Conjugated Polycyclic Hydrocarbons with Open-Shell Singlet Ground State: Nonazethrene versus Nonacene. *Journal of the American Chemical Society* **2016**, *138*, 10323–10330.

3 Korytár, R.; Xenioti, D.; Schmitteckert, P.; Alouani, M.; Evers, F. Signature of the Dirac cone in the properties of linear oligoacenes. *Nature Communications* **2014**, *5*, 5000.

4 van Setten, M. J.; Xenioti, D.; Alouani, M.; Evers, F.; Korytár, R. Incommensurate Quantum Size Oscillations of Oligoacene Wires Adsorbed on Au(111). *The Journal of Physical Chemistry C* **2019**, *123*, 8902–8907.

5 Piquero-Zulaica, I.; Garcia-Lekue, A.; Colazzo, L.; Krug, C. K.; Mohammed, M. S. G.; Abd El-Fattah, Z. M.; Gottfried, J. M.; de Oteyza, D. G.; Ortega, J. E.; Lobo-Checa, J. Electronic Structure Tunability by Periodic meta-Ligand Spacing in One-Dimensional Organic Semiconductors. *ACS Nano* **2018**, *12*, 10537–10544.
