# Supplementary figures and images for: Metallic bands in chevron-type polyacenes
Source: RSC Adv. 2020 Sep 14;10(56):33844–50. doi: 10.1039/d0ra06007k (PMC9528856; doi:10.1039/d0ra06007k)

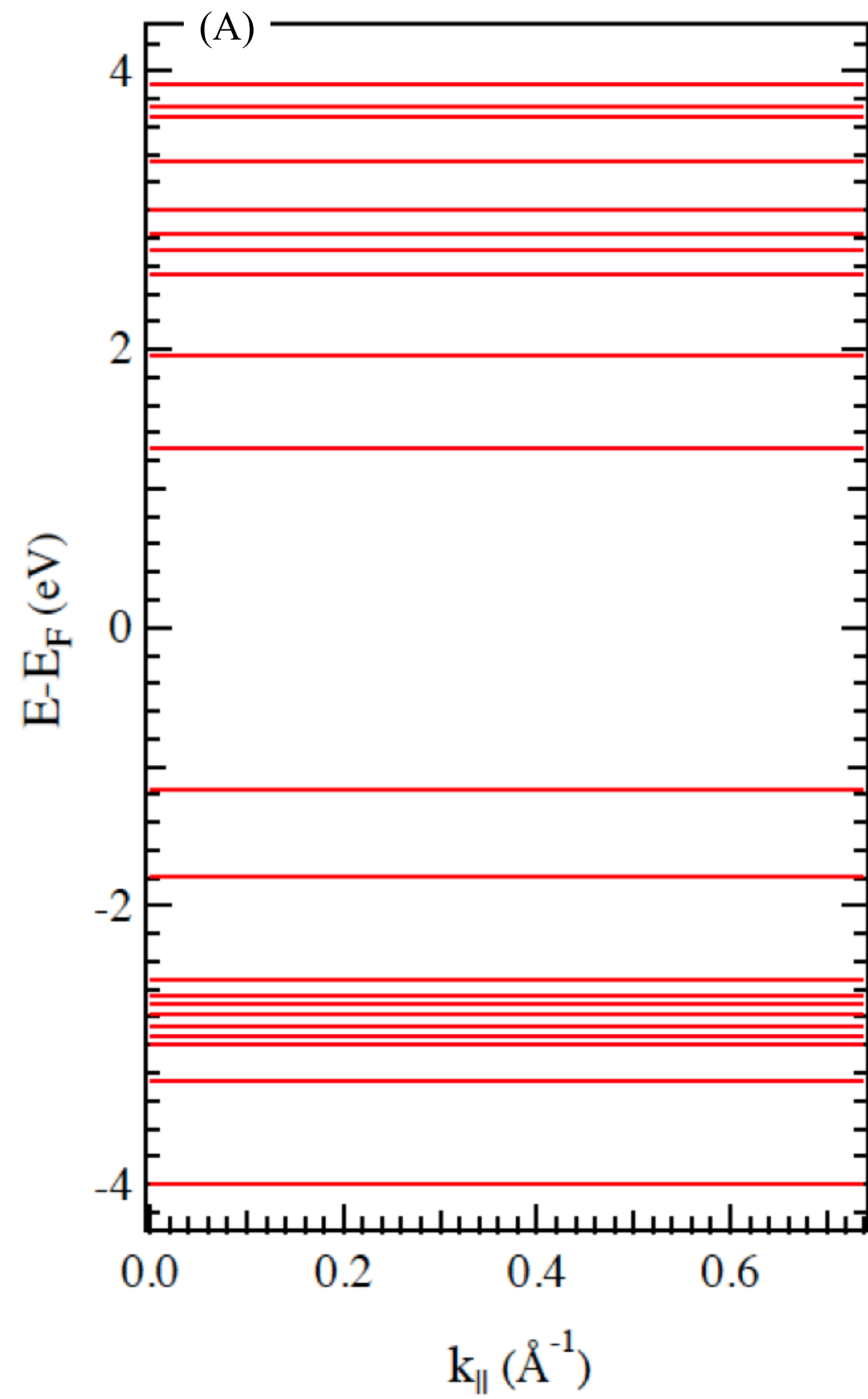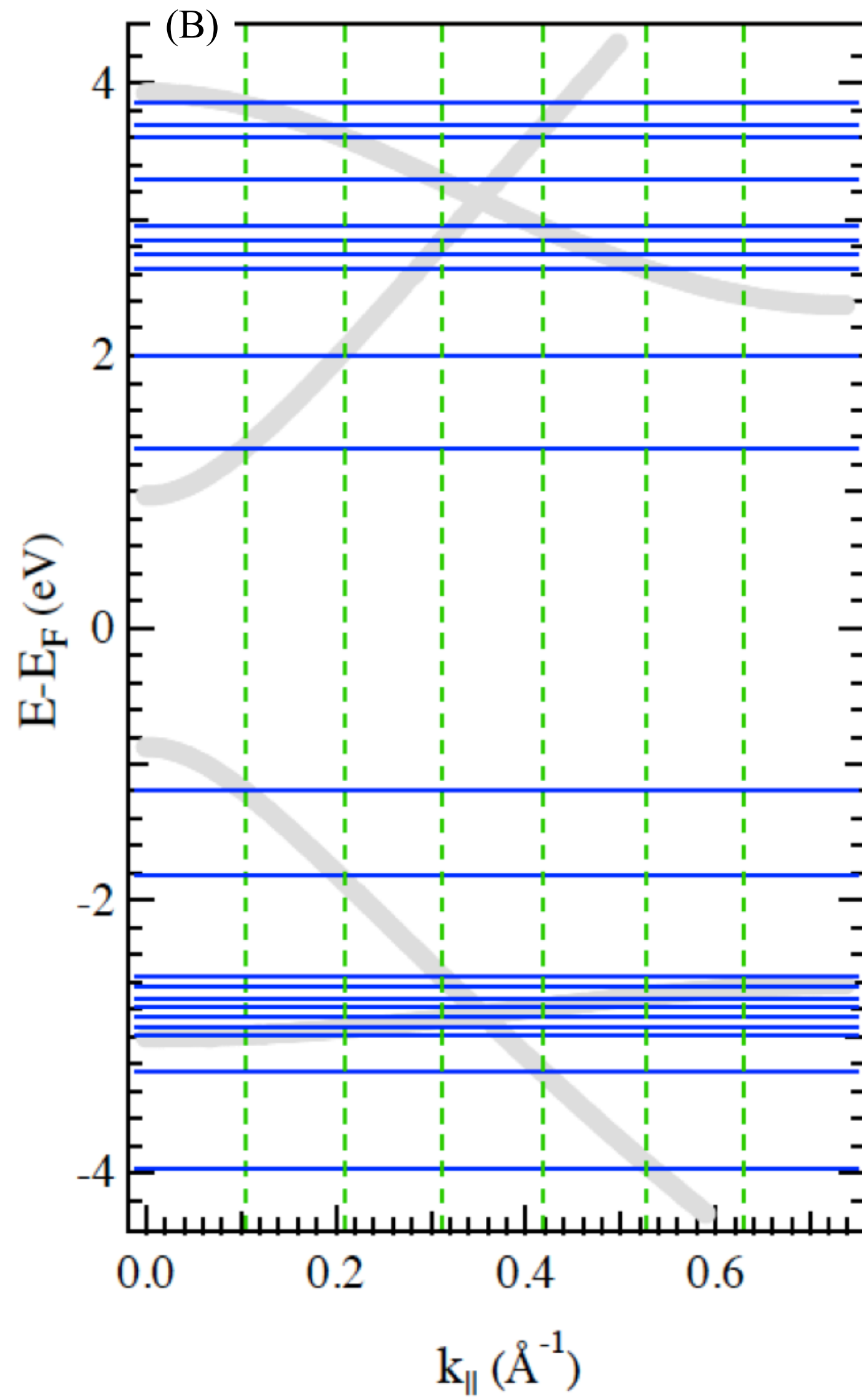

Supplement: RA-010-D0RA06007K-s002 [file RA-010-D0RA06007K-s002.zip › SI/Fig_S1.pdf]

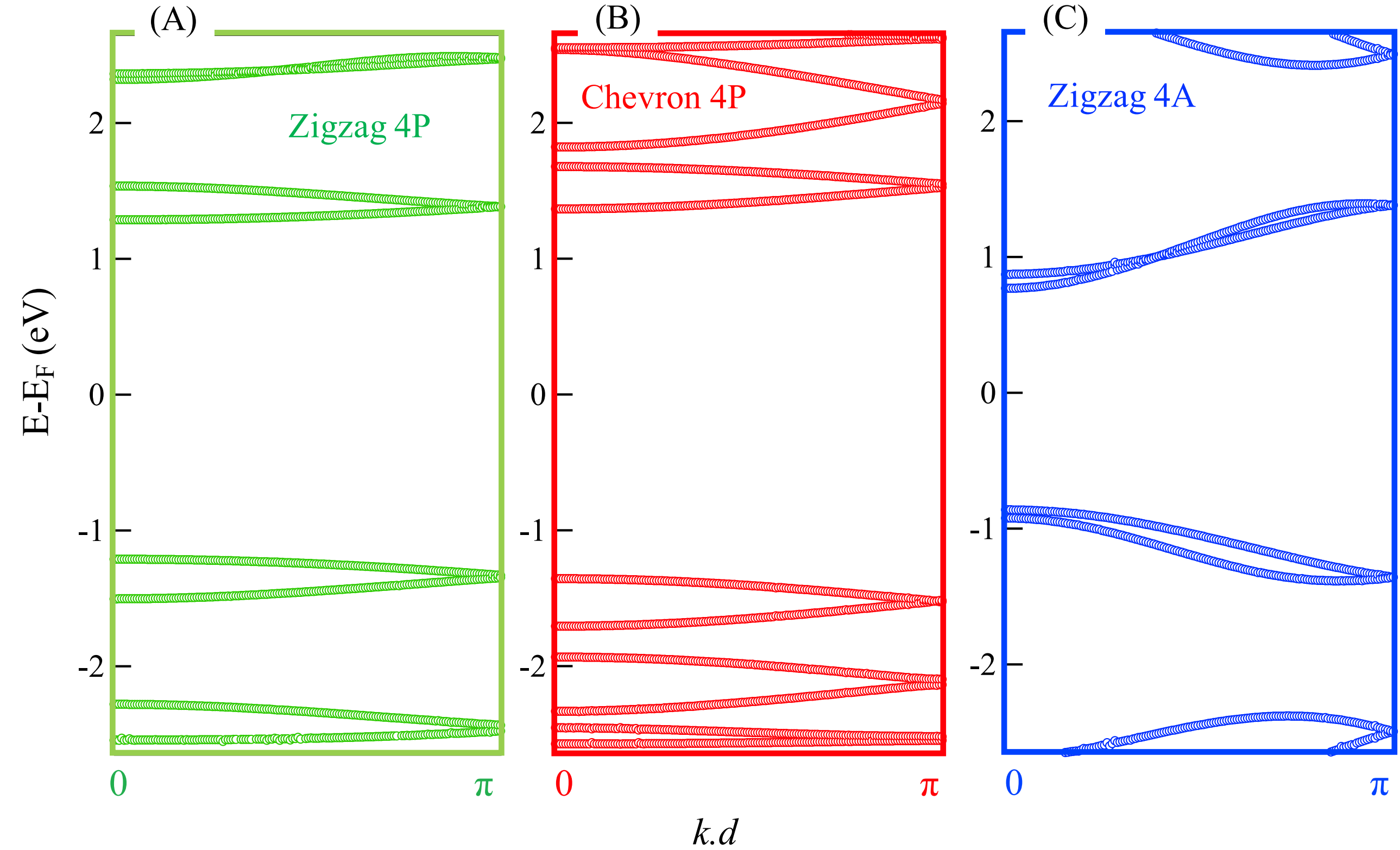

Supplement: RA-010-D0RA06007K-s002 [file RA-010-D0RA06007K-s002.zip › SI/Fig_S4.pdf]

LDOS (Arb.units)

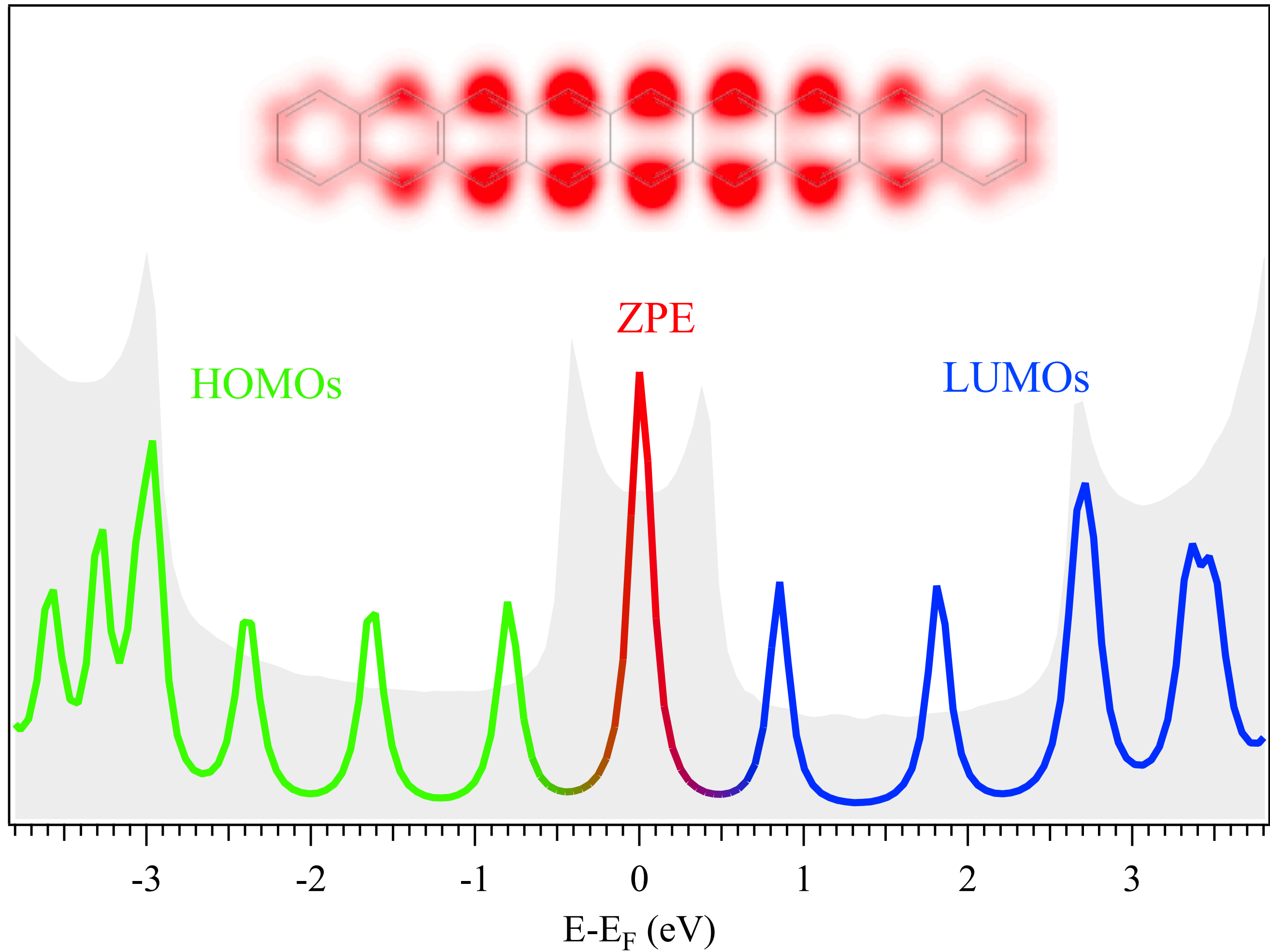

Supplement: RA-010-D0RA06007K-s002 [file RA-010-D0RA06007K-s002.zip › SI/Fig_S2_New.pdf]

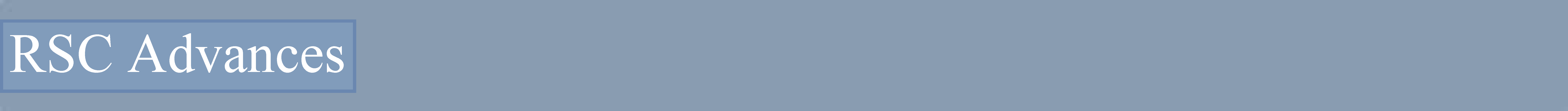

Supplement: RA-010-D0RA06007K-s002 [file RA-010-D0RA06007K-s002.zip › SI/head_foot/header_bar2.png]

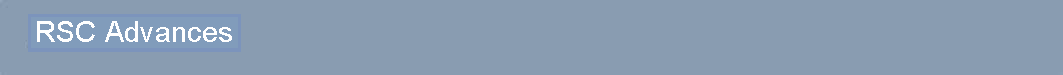

Supplement: RA-010-D0RA06007K-s002 [file RA-010-D0RA06007K-s002.zip › SI/head_foot/header_bar2.tiff]

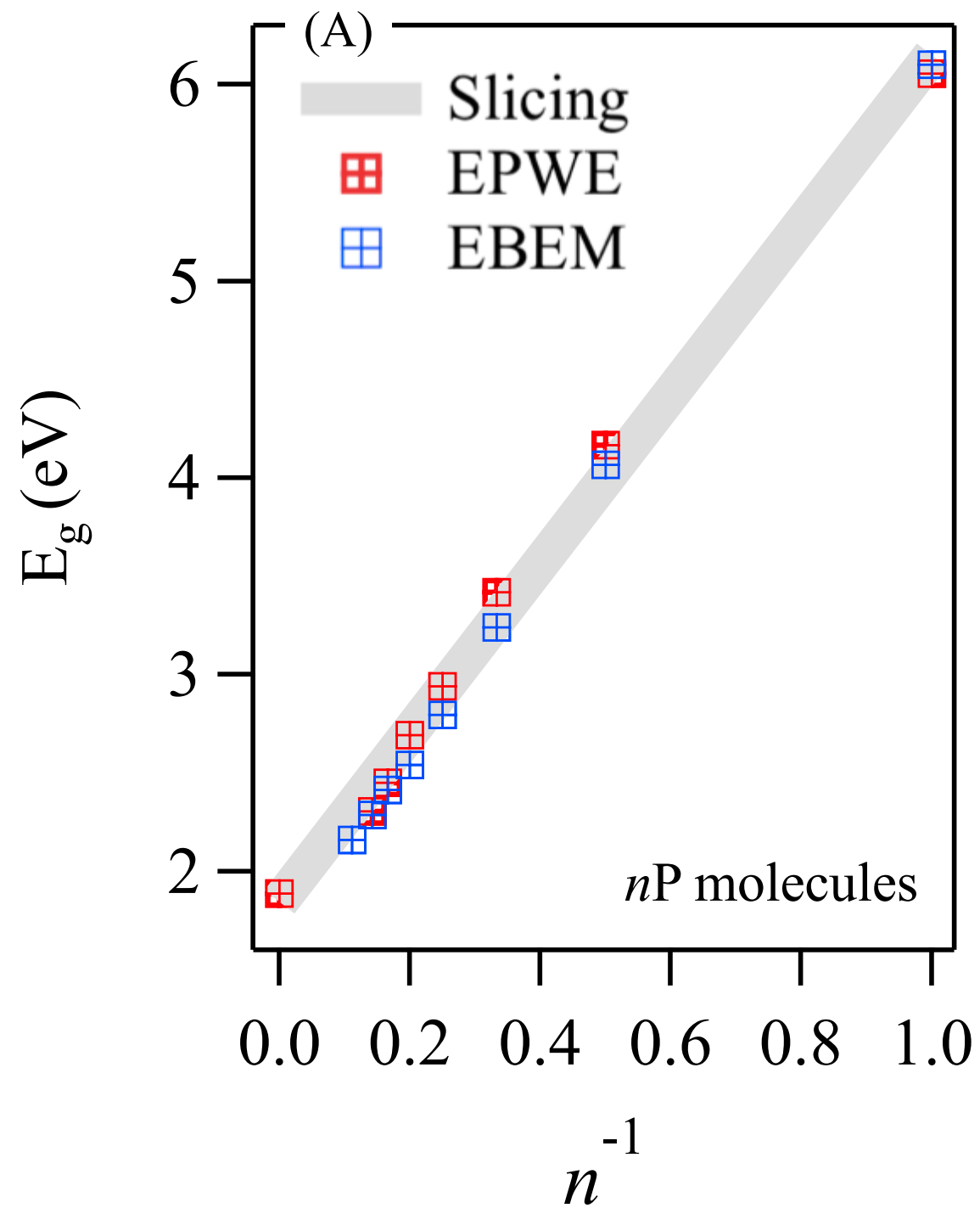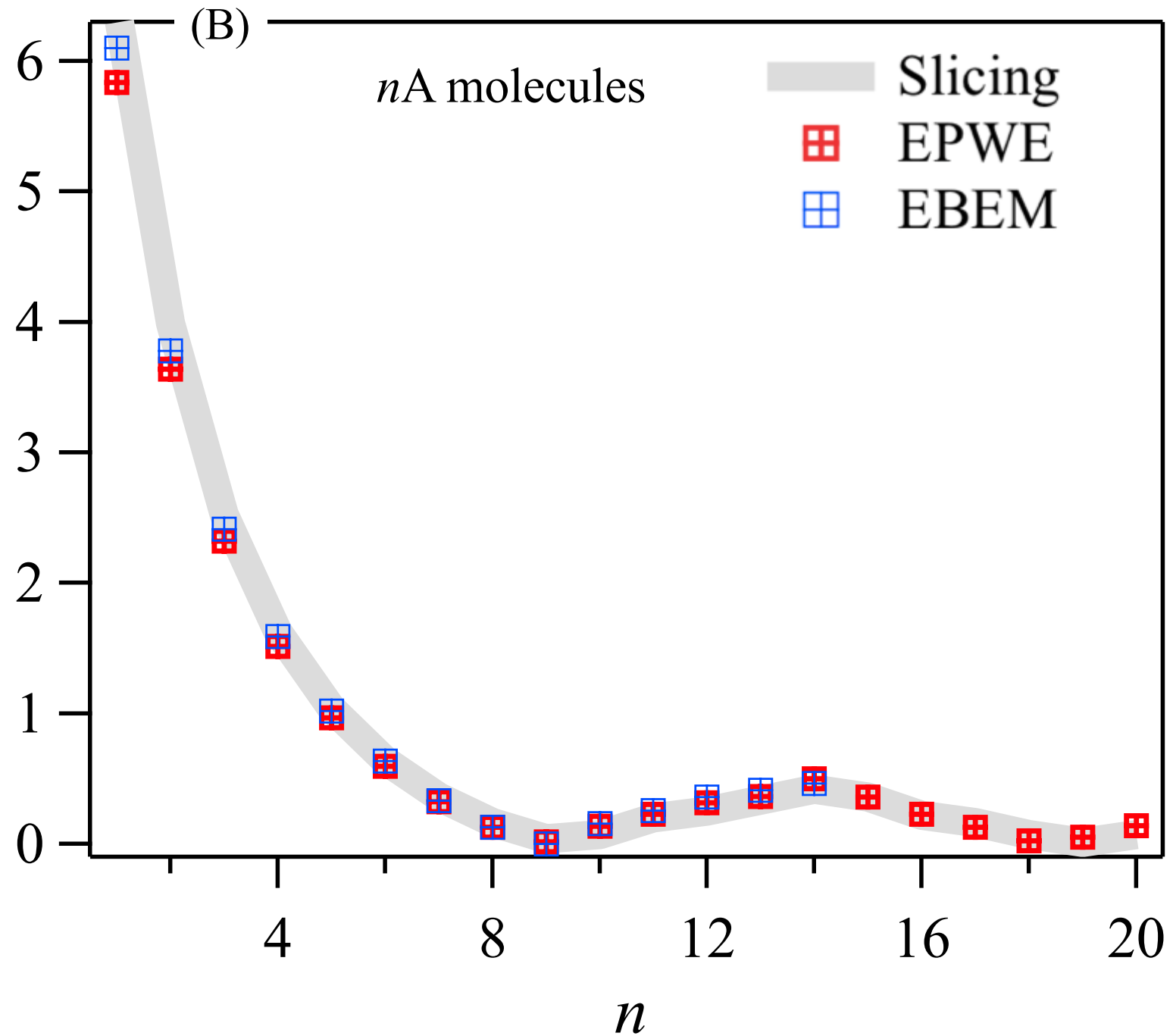

Supplement: RA-010-D0RA06007K-s002 [file RA-010-D0RA06007K-s002.zip › SI/Fig_S3_New.pdf]
